# Facet controlled growth mechanism of SnO_2_ (101) nanosheet assembled film via cold crystallization

**DOI:** 10.1038/s41598-021-90939-4

**Published:** 2021-05-28

**Authors:** Yoshitake Masuda

**Affiliations:** National Institute of Advanced Industrial Science and Technology (AIST), 2266-98 Anagahora, Shimoshidami, Moriyama-ku, Nagoya, 463-8560 Japan

**Keywords:** Materials chemistry, Surface chemistry

## Abstract

Cold crystallization of SnO_2_ was realized in aqueous solutions, where crystal growth was controlled to form SnO_2_ (101) nanosheet assembled films for devices such as chemical sensors. The nanosheets grew directly on a fluorine-doped tin oxide substrate without a seed layer or a buffer layer. The nanosheets had a thickness of 5–10 nm and an in-plane size of 100–1600 nm. Moreover, the large flat surface of the (101) facet was metastable. The thickness of the SnO_2_ (101) nanosheet assembled film was approximately 800 nm, and the film had a gradient structure that contained many connected nanosheets. TEM results revealed that the predominate branch angles between any two connected nanosheets were 90° and 46.48°, corresponding to type I and type II connections, respectively. These connections were consistent with the calculations based on crystallography. Crystallographic analysis clarified the characteristic crystal growth of the SnO_2_ (101) nanosheet assembled film in the aqueous solution. Furthermore, we demonstrate that the metastable (101) facet can be exploited to control the rate of crystal growth by adjusting the etching condition.

## Introduction

Metal oxide nanostructures have been developed on fluorine-doped tin oxide (FTO) substrates for various devices such as chemical sensors^1–3^. In particular, a photoelectric conversion molecular sensor was recently proposed by combining a metal oxide nanostructured film with FTO^4–7^. The mechanism of the sensor was based on photoelectric conversion and the antigen–antibody reaction. The FTO substrate possessed high transparency, which was essential for the transmission of excitation light. Moreover, its high electrical conductivity of 9.3–9.7 Ω/square (ohm/square) was suitable for producing photocurrent. Compared to an indium-doped tin oxide (ITO) transparent conductive film, the FTO film had a large surface roughness and a high surface area, which were effective at increasing the adsorption of dye-labeled monoclonal antibody. In addition, FTO was effective at facilitating specific adsorption for this sensor. Therefore, FTO was selected as a substrate in this study.

A photoelectric conversion molecular sensor has been developed using TiO_2_ nanostructured films on a FTO substrate^4,5^. The nanostructured anatase TiO_2_ film was deposited on the FTO substrate in an aqueous solution^4^. The film was an assembly of acicular TiO_2_ nanocrystals, where the crystals grew along the *c*-axis to produce needles that oriented in a direction perpendicular to the substrate. The nanostructured TiO_2_ film showed high dye adsorption capacity, which was approximately 6 times higher than that of a particulate film constructed of TiO_2_ nanoparticles (commercial TiO_2_ P25, Degussa)^4^. The nanostructured TiO_2_ film, covered with dye-labeled proteins^5^, showed high photoluminescence and a photocurrent of 4 nA under an excitation light of 633 nm in wavelength. Thus, the combination of a nanostructured TiO_2_ film and FTO has high potential in molecular sensor applications.

The SnO_2_ (101) nanosheet assembled film has been applied in a molecular sensor^6^. The high surface area and internal nanospace of the SnO_2_ (101) nanosheet assembled films were effective at enhancing the performance of the molecular sensor. Additionally, SnO_2_ had the advantage of suppressing nonspecific adsorption of molecules. The photoluminescence intensity from the SnO_2_ (101) nanosheet assembled film was up to 16 times larger than that from the bare FTO substrate^6^. A high photocurrent of 5.5 × 10^−6^ A and a high signal-to-noise ratio of 29 were achieved^6^.

The SnO_2_ (101) nanosheet assembled film has also been applied in a cancer sensor^7^. The film was modified with dye-labeled monoclonal antibody that reacted with human alpha fetoprotein in the blood serum of a hepatocellular cancer patient. Photoluminescence and photocurrent were detected from the film under red light excitation. The photoluminescence was 2600 times greater than that from the SnO_2_ (101) nanosheet assembled film with unlabeled prostate specific antigen. The photocurrent reached 603.7 nA. Therefore, the sensor can be used for the detection of hepatocellular cancer.

Moreover, cancer marker gas sensing has been demonstrated with the SnO_2_ (101) nanosheet assembled film^8^. The (101) crystal facet of the SnO_2_ nanosheet was utilized as a sensitive membrane to adsorb and oxidize 1-nonanal molecules. The sensitivity (Ra/Rg) was evaluated from the sensor resistance in air (Ra) and sensor resistance in a target gas (Rg). A low concentration of the 1-nonanal gas (550 ppb) was clearly detected with a high sensitivity of over 10, and the sensitivity increased with increasing gas concentration. The SnO_2_ (101) nanosheets also showed high sensitivity for detecting lung cancer markers.

The SnO_2_ (101) nanosheet assembled film has been applied to H_2_ gas sensing^9^. The sensitivity increased with increasing H_2_ concentration in the range of 5–5000 ppm. Specifically, the sensitivities at 5, 50, 500, and 5000 ppm were 1.6, 3.2, 12.8, and 31.8, respectively. Indoor air containing various components and moisture was used for the evaluation, and the H_2_ sensor was demonstrated to work in real environments. The SnO_2_ nanosheet assembled films also showed high potential for alkene gas sensing^10^. The SnO_2_ nanosheet showed high sensor response to alkene gases such as C_2_H_4_, C_3_H_6_, or 1-C_4_H_8_ compared with alkane gases such as CH_4_, C_2_H_6_, C_3_H_8_, or n-C_4_H_10_. Gas selectivity is an important property of the sensor. Alkene/alkane gas selectivity of the SnO_2_ nanosheet was higher than that of a typical commercial sensor (TGS2602, FIGARO). Additionally, sensor response increased with increasing molecule weight of alkene gases or alkane gases. Alkene/alkane gas selectivity is one of the characteristics of the (101) crystal facet of the SnO_2_ nanosheet. The SnO_2_ nanosheet showed sensor response of 7.5 to H_2_ gas^11^. It was more than two times higher than that of the SnO_2_ nanoparticle (response: 3.0), which is covered with the most stable (110) crystal facets. The (101) crystal facet and nanosheet structure were effective for H_2_ gas sensing. Additionally, H_2_/CH_4_ gas selectivity of the SnO_2_ nanosheet was 5.8 (= 7.5/1.3). It was more than two times higher than that of the SnO_2_ nanoparticle (H_2_/CH_4_ gas selectivity: 2 = 3.0/1.5). The 90% sensor response time of the SnO_2_ nanosheet was 6 s for H_2_ gas. It was three times faster than that of the SnO_2_ nanoparticle (response time: 18 s). Response time of the SnO_2_ nanosheet for CH_4_ gas was 18 s, which was also faster than that of the SnO_2_ nanoparticle (response time: 24 s). Fast response is attractive for sensor applications. It is one of the advantages of the SnO_2_ nanosheet.

Theoretical calculations of the (110) or (101) facet revealed the effect of different surfaces on the device properties. Zakaryan et al*.* studied the adsorption process of CO on the (110), (100), (101), and (001) surfaces of SnO_2_ with density functional theory (DFT) calculations^12^. The electronic density of states combined with a Bader charge analysis showed that the (101) and (001) surface orientations gathered more electrons than the other orientations, such as (110). They concluded that the (101) or (001) surface of SnO_2_ was a better platform for CO gas sensing. Jiang et al*.* conducted theoretical calculations of the SnO_2_ (110), (101), and (221) facets for NO and NO_2_ gas sensing^13^. The optimized structures of the NO molecule adsorbed on the (110), (101), and (211) facets were obtained. The total energy was calculated with DFT to investigate the adsorption of the molecules. The exchange and correlation energies were calculated with the Perdew–Wang (PW) functional with the generalized gradient approximation (GGA), and a double numerical plus polarization basis set was used (DNP). The configuration of the adsorbed NO molecule was more stable in the nitrogen orientation than in the oxygen orientation on the (101) crystal surface. The adsorption energy (binding energy) of NO on the (110) facet was the lowest and that on the (211) facet was the highest. They concluded that the (101) facet was the most beneficial among the three crystal surfaces to both NO and NO_2_ gas detection based on the determined adsorption energies and electron transfer. Abokifa et al*.* investigated the sensing mechanism of SnO_2_ for ethanol and acetone at room temperature with first principles DFT calculations and ab initio molecular dynamics (AIMD) simulations^14^. They concluded that both the ethanol and acetone molecules had a stronger interaction with the pre-adsorbed O_2_^−^ species on the reduced (101) surface than on the reduced (110) surface at room temperature. The most cited sensing mechanism model was the interaction with the ionosorbed O^−^ species that possessed high activity for oxidizing target gas molecules. However, the less active superoxide molecule (O_2_^−^) constituted the majority of the pre-adsorbed oxygen species on the SnO_2_ surface at room temperature. Both the ethanol and acetone molecules could not exothermically bind at the pre-occupied vacancy location and showed no interactions with the pre-adsorbed O_2_^−^ species on the reduced (110) surface. On the other hand, the oxygen vacancy site simultaneously accommodated the O_2_^−^ species together with the ethanol or acetone molecule on the reduced (101) surface, which forced the pre-adsorbed O_2_^−^ species to release the minor charge back to the surface. AIMD simulations demonstrated that both molecules first preferred to bind at the unoccupied oxygen vacancy sites (i.e., at the undercoordinated Sn4c). Once all the oxygen vacancy sites were filled with pre-adsorbed oxygen from the ambient atmosphere, the target molecule tended to momentarily share the preoccupied vacancy with the pre-adsorbed oxygen molecule before eventually binding at a surface Sn5c site. Therefore, the adsorption configuration resembled stoichiometric surfaces. They also reported that adsorption was generally stronger for ethanol than for acetone due to the bipolar nature of the hydroxyl group (on ethanol) that interacted with the surface via two distinct charge transfer modes. The results suggested that the sensing mechanism of SnO_2_ for polar VOCs at room temperature can be explained by their direct adsorption on the surface rather than through their oxidation by ionosorbed oxygen species. Feng et al*.* investigated the relationship between morphology and the humidity sensing property of SnO_2_ with DFT calculations^15^. The calculations indicated that the {101} facets adsorbed more water molecules than the {110} facets. This result was consistent with the experimental data in which the nanosensor based on three-dimensional (3D) hierarchical SnO_2_ dodecahedral nanocrystals (DNCs) exhibited superior humidity-sensing performance including fast response and recovery times, narrow hysteresis loop, high sensitivity, great linearity response, and good stability compared with the 3D hierarchical SnO_2_ nanorods (NRs) and SnO_2_ nanoparticles (NPs). They concluded that the enhanced sensing properties of the DNCs were attributed to the peculiar 3D open nanostructures and high chemical activity of the exposed {101} facet. In summary, the 3D open nanostructures can promote the penetration and diffusion of water molecules, and the exposed {101} facets can improve the adsorption ability of water molecules.

The SnO_2_ (101) nanosheet assembled films have been applied to molecular sensors and gas sensors as described above. Their characteristic (101) crystal facets and nanosheet morphology were reported to possess novel functions. However, crystallographic analysis of the SnO_2_ (101) nanosheet assembled film has not been reported yet. This article is focused on analyzing the crystal growth and morphology of the SnO_2_ (101) nanosheet assembled film. The growth mechanism and crystallographic models of the nanosheet are discussed.

## Experimental procedure

### Crystallization of the SnO_2_ (101) nanosheet assembled film on the FTO substrate

The SnO_2_ (101) nanosheet assembled film was formed on the FTO substrate following the conventional method used to make photocurrent conversion type molecular sensors. The FTO substrate (SnO_2_:F, Asahi Glass Co. Ltd., 26 × 50 × 1.1 mm) was blown by air to remove dust and was then exposed to vacuum ultraviolet (VUV) light for 10 min using a low-pressure mercury lamp (PL16-110, SEN Lights Co., air flow, 100 V, 200 W) At a distance of 10 mm from the lamp, the power densities were 14 mW/cm^2^ and 18 mW/cm^2^ for wavelengths of 184.9 nm and 253.7 nm, respectively. The FTO substrate was covered with paper, stacked, and stored in air at the factory. The FTO surface was hydrophobic with a water contact angle of 96° and was modified with VUV exposure to become a super hydrophilic surface with a contact angle of less than 1°. This was because the VUV exposure decomposed the attached organic molecules that were present in very small amounts on the surface of the FTO substrate. The super hydrophilization effect of VUV exposure has been reported for studies on the modification of a hydrophobic organic monolayers into super hydrophilic hydroxyl groups^16,17^. Hydrophilic surfaces with hydroxyl groups were reported to be very effective at the nucleation and crystal growth of metal oxide in an aqueous solution. Therefore, the FTO substrate was subjected to a treatment that produced super hydrophilic surfaces.

SnF_2_ (CAS 7783-47-3, FW: 156.71, FUJIFILM Wako Pure Chemical Corporation, No. 202-05485, purity 90.0%) was used as the raw material for the SnO_2_ crystals. A total of 200 mL of distilled water was heated to 90 °C in a polypropylene container using a drying furnace (DKN402, Yamato Scientific Co. Ltd.). SnF_2_ (870.6 mg) was dissolved in distilled water at 90 °C to a concentration of 25 mM. The FTO substrate was immersed in the middle of the solution with the bottom up, tilted at 15° to the upright. The solution was kept at 90 °C for 24 h. The FTO substrate was taken out of the solution, washed with running distilled water, and subjected to strong air blow. The SnO_2_ (101) nanosheet assembled film was crystallized on the FTO surface using this technique.

### Characterization of the SnO_***2***_ (101) nanosheet assembled film on the FTO substrate

The surface morphology of the SnO_2_ (101) nanosheet assembled film on the FTO substrate was observed with a field emission scanning electron microscope (FE-SEM; JSM-6335FM, JEOL Ltd.) The fractured cross-sectional morphology of the SnO_2_ (101) nanosheet assembled film was also observed with the FE-SEM after cutting the substrate. Samples for cross-sectional observation on a transmission electron microscope (TEM; FEI Tecnai Osiris) were prepared by the focused ion beam (FIB) micro sampling method at an accelerating voltage of 10–40 kV. Cross-sectional images of the substrates were obtained with the TEM operating at an accelerating voltage of 200 kV. A sample was prepared to observe the morphology from the direction perpendicular to the substrate. The sample piece was mechanically polished to approximately 20 μm from the glass substrate side. The sample was then thinned by Ar ion milling (liquid nitrogen cooling) at an accelerating voltage of ~ 3 keV. Plane images of the substrate were obtained with the TEM at an accelerating voltage of 200 kV. The crystalline phase was analyzed with an electron diffractometer (ED) installed on the TEM. The lattice spacing, crystal orientation, etc. were analyzed with electron diffraction (ED) and fast Fourier transform (FFT) images. High-angle annular dark field (HAADF) images were also obtained with the scanning TEM (STEM) mode. Elemental mapping images were obtained using an energy dispersive X-ray spectroscopy (EDS) unit integrated in the TEM. The STEM probe size was approximately 1 nm. The dwell time and collection time were 20 μs and 300 s, respectively. Count map images were obtained for tin, oxygen, and aluminum. The samples were fixed using resin containing oxygen, carbon, and aluminum.

## Results and discussion

### SEM observation of the SnO_***2***_ (101) nanosheet assembled film

**Figure 1:**
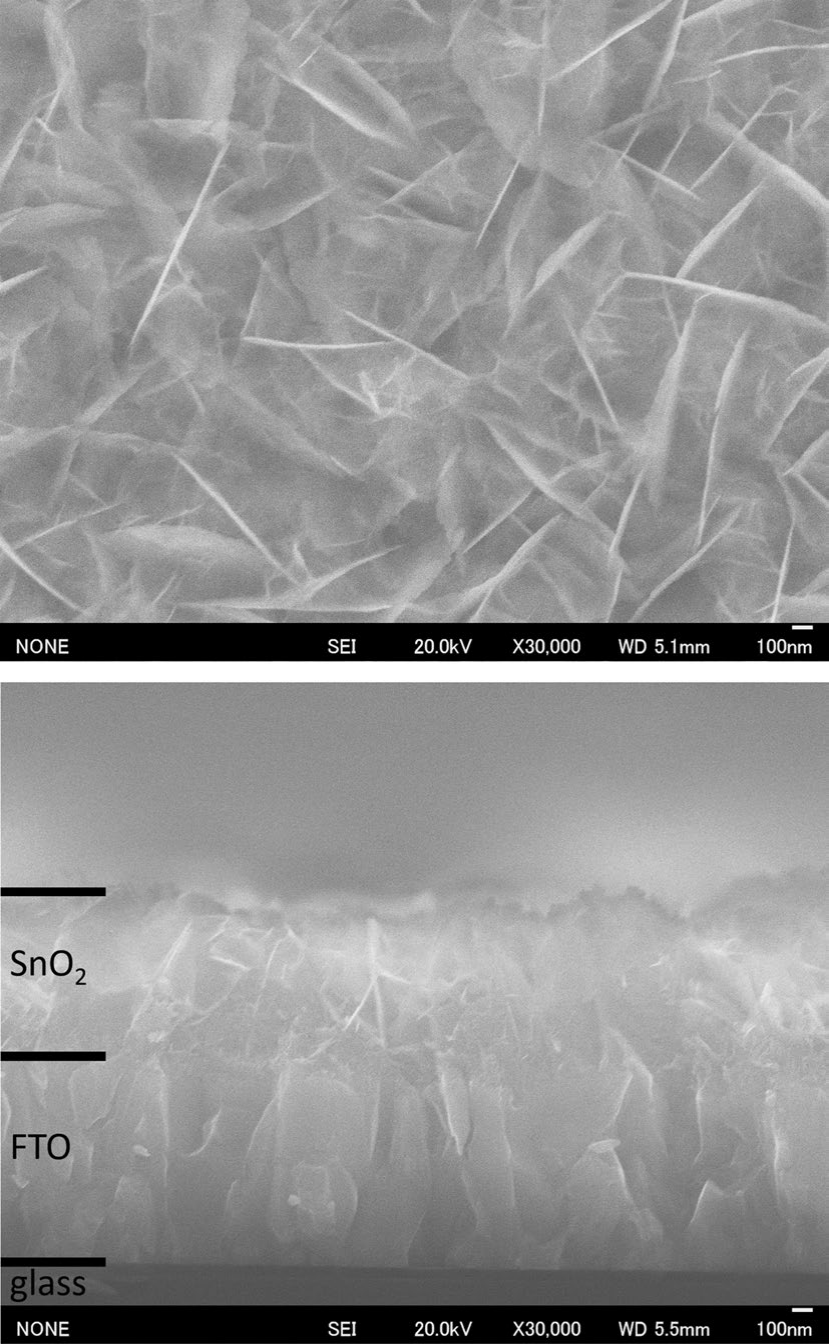
Surface (upper) and cross-sectional (lower) SEM images of the SnO_2_ (101) nanosheet assembled film.

The SnO_2_ (101) nanosheet assembled film was successfully fabricated on the entire surface of the FTO substrate (Fig. 1, upper image). The cross-sectional image showed that the thickness of the SnO_2_ nanosheet assembled film was approximately 800 nm (Fig. 1, lower image). The FTO layer formed on a glass substrate had a thickness of approximately 1000 nm. The SnO_2_ (101) nanosheets uniformly formed on the substrate had a thickness of 5–10 nm and an in-plane size of 100–1600 nm (Fig. 1, upper image). The reaction conditions such as temperature and ion concentration were uniform regardless of the location within the solution. The uniformity of the reaction conditions resulted in uniform nanosheet formation, which is one of the advantages of the solution process. In addition, film formation was realized at room temperature and atmospheric pressure in the aqueous solution with an open-air system. Therefore, the method used in this study can be applied to film formation on the meter size scale or to substrates with complicated shapes, such as uneven substrates, particles, fibers, or meshes. Furthermore, sintering at temperatures of the order of ~ 100 °C, which is usually required in the synthesis of ceramics, was unnecessary. Therefore, this method is applicable to film formation on low heat resistant plastics, carbon materials, metals, glasses, organic materials, biomaterials, etc. because of the room temperature process.

### Crystallographic analysis of the SnO_***2***_ (101) nanosheet assembled film with plane observed TEM images

**Figure 2:**
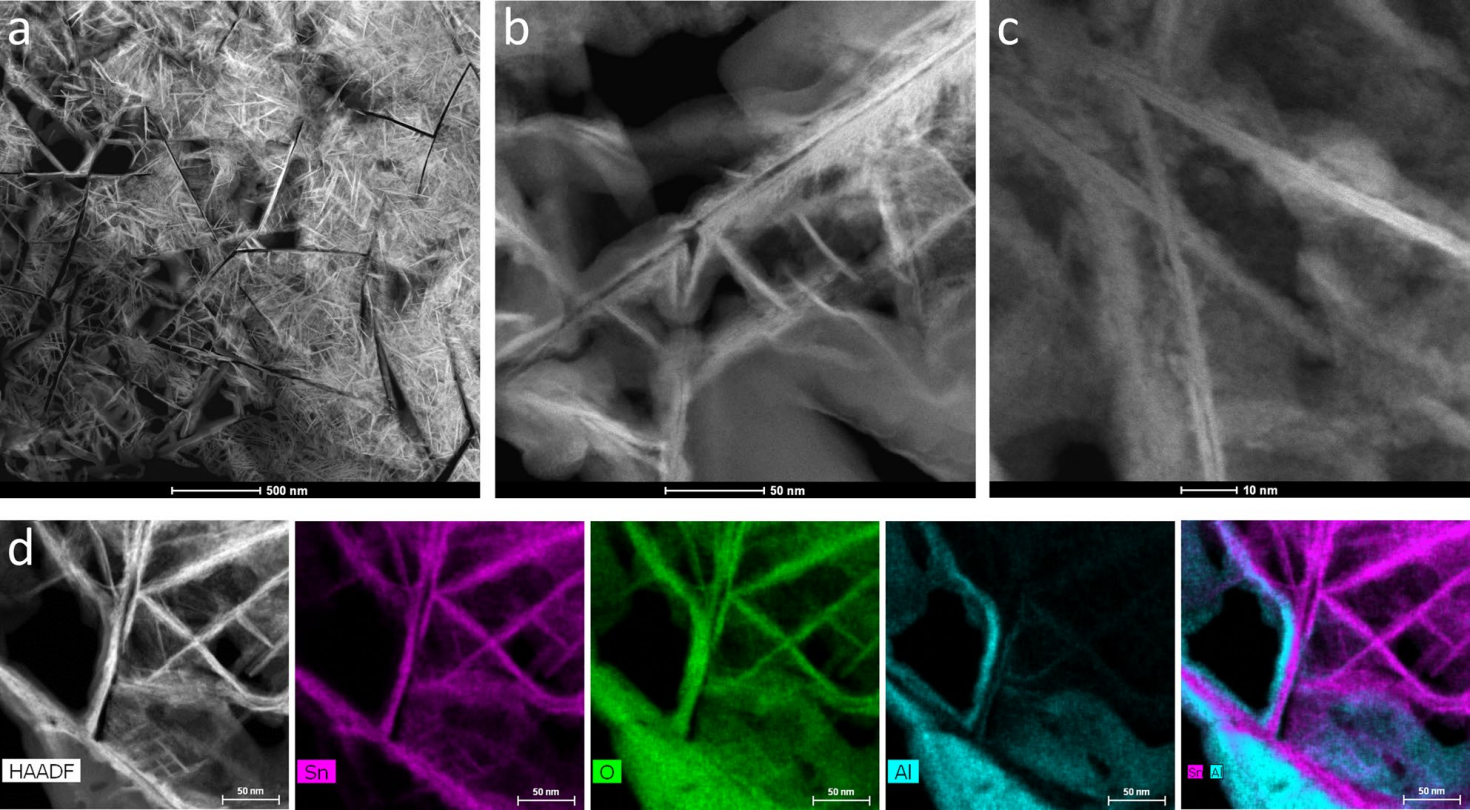
(**a**) Plane-observed HAADF-STEM image of the SnO_2_ (101) nanosheet assembled film. (**b**,**c**) Magnified image of an area of (**a**). (**d**) HAADF-STEM image and its EDS mapping images for Sn, O, Al, and Sn-Al.

Plane observed HAADF-STEM images of the SnO_2_ (101) nanosheet assembled film showed a network of the nanosheets (Fig. 2a). The nanosheets connected with each other to form a porous structure with inner nano/mirco-meter sized spaces; this characteristic nanosheet-assembled structure was produced by the crystal growth of SnO_2_. A large SnO_2_ (101) nanosheet with a thickness of 4 nm, an in-plane size of over 300 nm, and an aspect ratio of over 75 was observed at the center of the image (Fig. 2b). Nanosheets with a thickness of 4 nm, an in-plane size of 50 nm, and an aspect ratio of 13 grew from the large nanosheet. A large number of small nanosheets with a thickness of 2 nm, an in-plane size of 10 nm, and an aspect ratio of 5 also grew from the large nanosheet. A SnO_2_ (101) nanosheet with a thickness of 3 nm, an in-plane size of over 100 nm, and an aspect ratio of over 33 was observed at the upper area of the image (Fig. 2c). Two dark gray lines were observed in the light gray nanosheet, and these lines were parallel to the in-plane direction. The surface of the nanosheets contained smaller SnO_2_ crystals of 1–2 nm in size, which contributed to the characteristic surface of the SnO_2_ (101) nanosheet assembled film.

The angle between the connected nanosheets was either approximately 90° or 40°–50° (Fig. 2d, HAADF). Tin was detected in the nanosheets (Fig. 2d, Sn), oxygen was observed in the nanosheets and the adhesive glue on the nanosheets (Fig. 2d, O), and aluminum was observed in the adhesive (Fig. 2d, Al). The adhesive was used to fix the sample for the TEM experiments and remained on the nanosheets. The mapping image of tin and aluminum clearly showed their positions in the nanosheets and the adhesive (Fig. 2d, Sn–Al), and the EDS results indicated that the nanosheets contained tin and oxygen.

**Figure 3:**
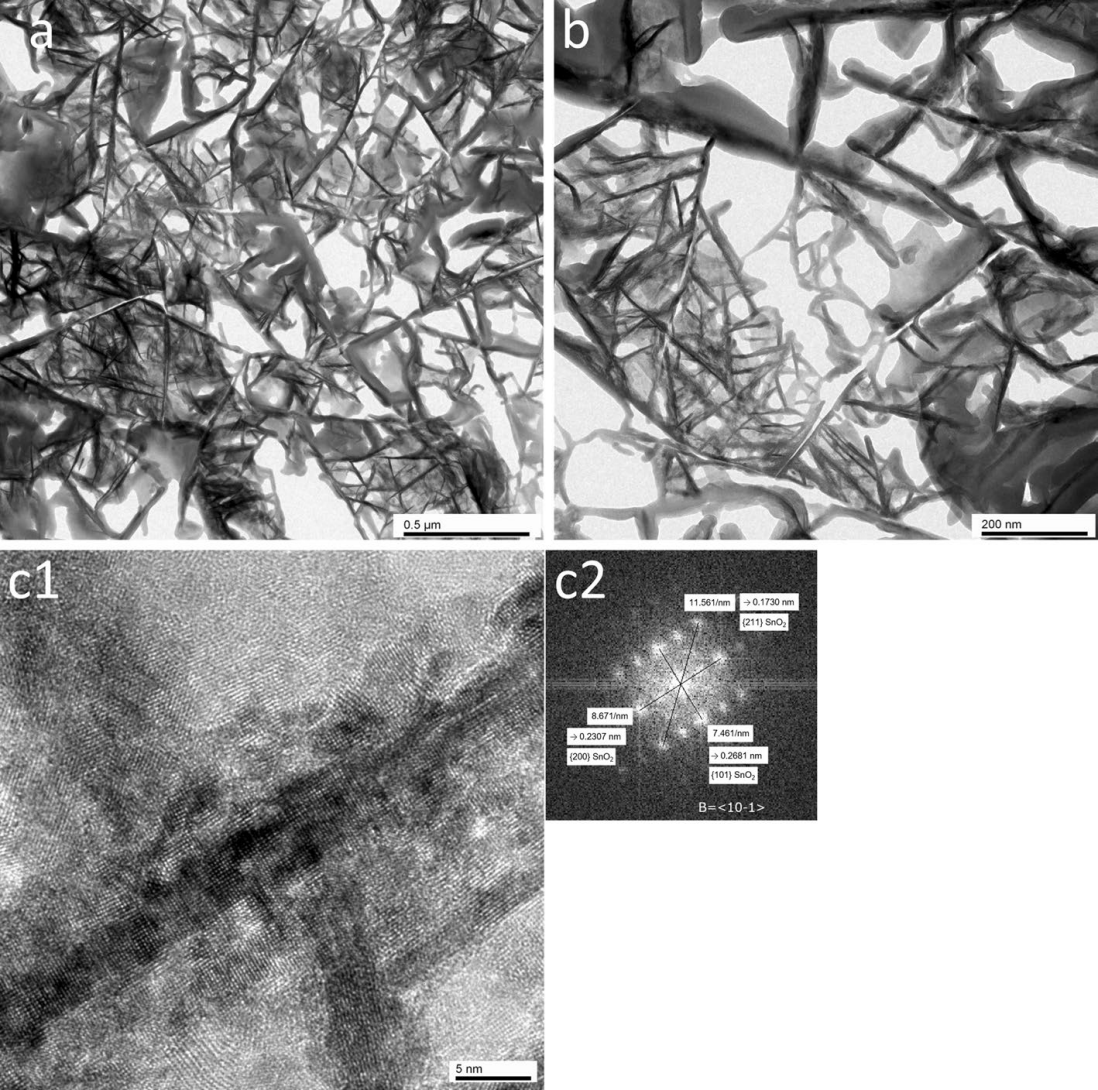
(**a**) Plane-observed TEM image of the SnO_2_ (101) nanosheet assembled film. (**b**) Magnified image of an area of (**a**). (**c1**) High-resolution plane-observed TEM image of the SnO_2_ (101) nanosheet. (**c2**) FFT image of (**c1**) showing the lattice spacings of {101}, {200}, and {211}.

A network of the SnO_2_ (101) nanosheets was also observed in the plane observed TEM images (Fig. 3a), which was formed by the dendritic growth of SnO_2_. The nanosheets connected with various angles. Impurities such as amorphous or spherical particles were not observed (Fig. 3b). However, the adhesive remained on the nanosheets. A high resolution TEM image of the nanosheet showed lattice fringes (Fig. 3c1), while an FFT image contained clear spots, which indicated that the nanosheet was a single crystal of SnO_2_ (Fig. 3c2). The lattice spacings were calculated from the spots in the FFT image. The lattice spacing of the plane parallel to the nanosheet was 0.2681 nm, and this plane was assigned to the {101} crystal pane. The FFT image indicated that the nanosheet had a (101) crystal facet, which was consistent with previous studies in which the (101) facet of the SnO_2_ nanosheet was observed^10,11,18,19^. The lattice spacing of the crystal plane perpendicular to the nanosheet was 0.2307 nm, and this plane was assigned to the {200} crystal pane of SnO_2_. The stacking direction of the {200} crystal planes was the *a*-axis of SnO_2_. The lattice spacing of the crystal plane at an angle with the nanosheet was 0.1730 nm, and this plane was assigned to the {211} crystal plane of SnO_2_.

**Figure 4:**
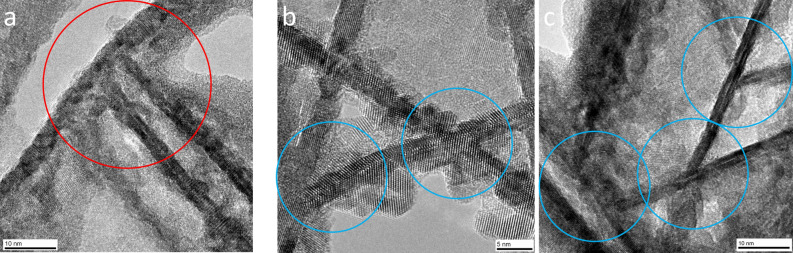
(**a**) High-resolution plane-observed TEM image of the SnO_2_ nanosheets connected with a branch angle of 90° (type I). (**b**,**c**) High-resolution plane-observed TEM image of the SnO_2_ nanosheets connected with a branch angle of 40°–50° (type II).

Some SnO_2_ nanosheets grew from other SnO_2_ nanosheets by dendritic growth and connected with a branch angle of 90° (red circle, type I) (Fig. 4a). The crystal structure and growth direction were affected by the branch angle. Notably, the SnO_2_ nanosheets grew directly from other SnO_2_ nanosheets without the appearance of an amorphous layer, impurity layer, or cracks at the interface. This growth feature contributed to the high mechanical strength, high electrical conductivity, high thermal conductivity, etc. of the film. In addition, some SnO_2_ nanosheets grew from other SnO_2_ nanosheets with a branch angle of 40°–50° (blue circle, type II) (Fig. 4b,c).

**Figure 5:**
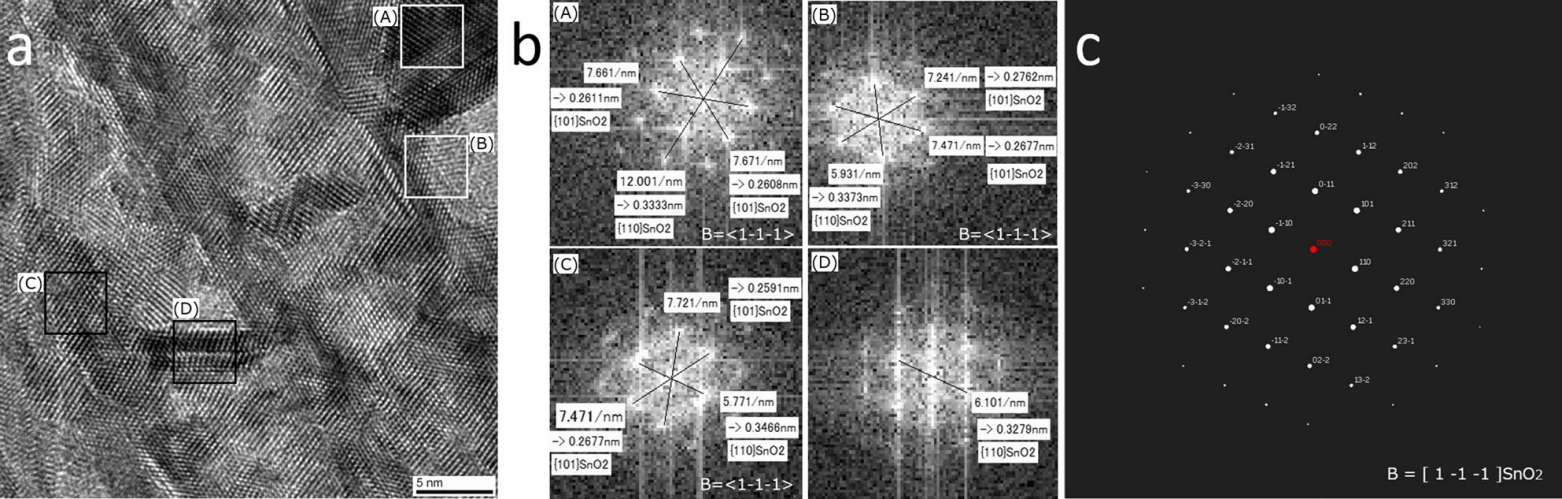
(**a**) High-resolution plane-observed TEM image of the SnO_2_ nanosheets connected with a branch angle of 90° (square A and B) or 40°–50° (square C and D). (**b**) FFT images of A, B, C, or D calculated from the TEM images in square A, B, C, or D, respectively. (**c**) Calculation of selected area electron diffraction (SAED) pattern from the direction of B =  < 1 − 1 − 1 > of SnO_2_.

The TEM images (Fig. 4) clearly showed the interface structure of the nanosheets. However, the TEM observation direction did not precisely align with the direction of the connected interface line of the nanosheets in these figures. In other words, the observation direction was not parallel to both of the connected nanosheets. The branch angles were evaluated from the TEM image, which was further observed from B = <1 − 1 − 1> of SnO_2_, as shown in Fig. 5. Three large nanosheets were observed in parallel from the upper left to the lower right of Fig. 5a. The FFT images of A, B, C, and D (Fig. 5b) were calculated from the TEM images in squares A, B, C, and D (Fig. 5a), respectively. Squares B and C were on one of the three large nanosheets. Square A was on the nanosheet that connected with one of the three large nanosheets containing square B; their branch angle was 90° (Fig. 5a). Square D was on the nanosheet that connected with one of the three large nanosheets containing square C; their branch angle was 40°–50° (Fig. 5a). The FFT images were analyzed to calculate the selected area electron diffraction (SAED) pattern from the direction of B = <1 − 1 − 1> of SnO_2_ (Fig. 5c). Thus, the lattice spacings of the nanosheets that were parallel to the large nanosheets in FFT B and C were respectively 0.2762 nm (Fig. 5b, B) and 0.2677 nm (Fig. 5b, C). They were assigned to the {101} crystal plane of SnO_2_ to indicate that the nanosheets had a (101) facet. The lattice spacings of 0.2677 nm and 0.3373 nm were assigned respectively to the {101} and {110} crystal plane of SnO_2_ (in FFT B). The lattice spacings of 0.2591 nm and 0.3466 nm were assigned respectively to the {101} and {110} crystal plane of SnO_2_ (in FFT C). They also provided information on the connections of the nanosheets.

The SnO_2_ nanosheet that connected with the large nanosheet with a branch angle of 90° is shown in square A. The lattice spacing of the nanosheet that was parallel to the nanosheets in FFT A was 0.2608 nm (Fig. 5b, A). It was assigned to the {101} crystal plane of SnO_2_ to indicate that the nanosheets had a (101) facet. The lattice spacings of 0.2611 nm and 0.3333 nm were assigned respectively to the {101} and {110} crystal plane of SnO_2_ (in FFT A). On the other hand, the SnO_2_ nanosheet that connected with the large nanosheet with a branch angle of 40°–50° is shown in square D. The lattice spacing of the nanosheet that was parallel to the nanosheets in FFT D was 0.3279 nm (Fig. 5b, D). It was assigned to the {110} crystal plane of SnO_2_.

**Figure 6:**
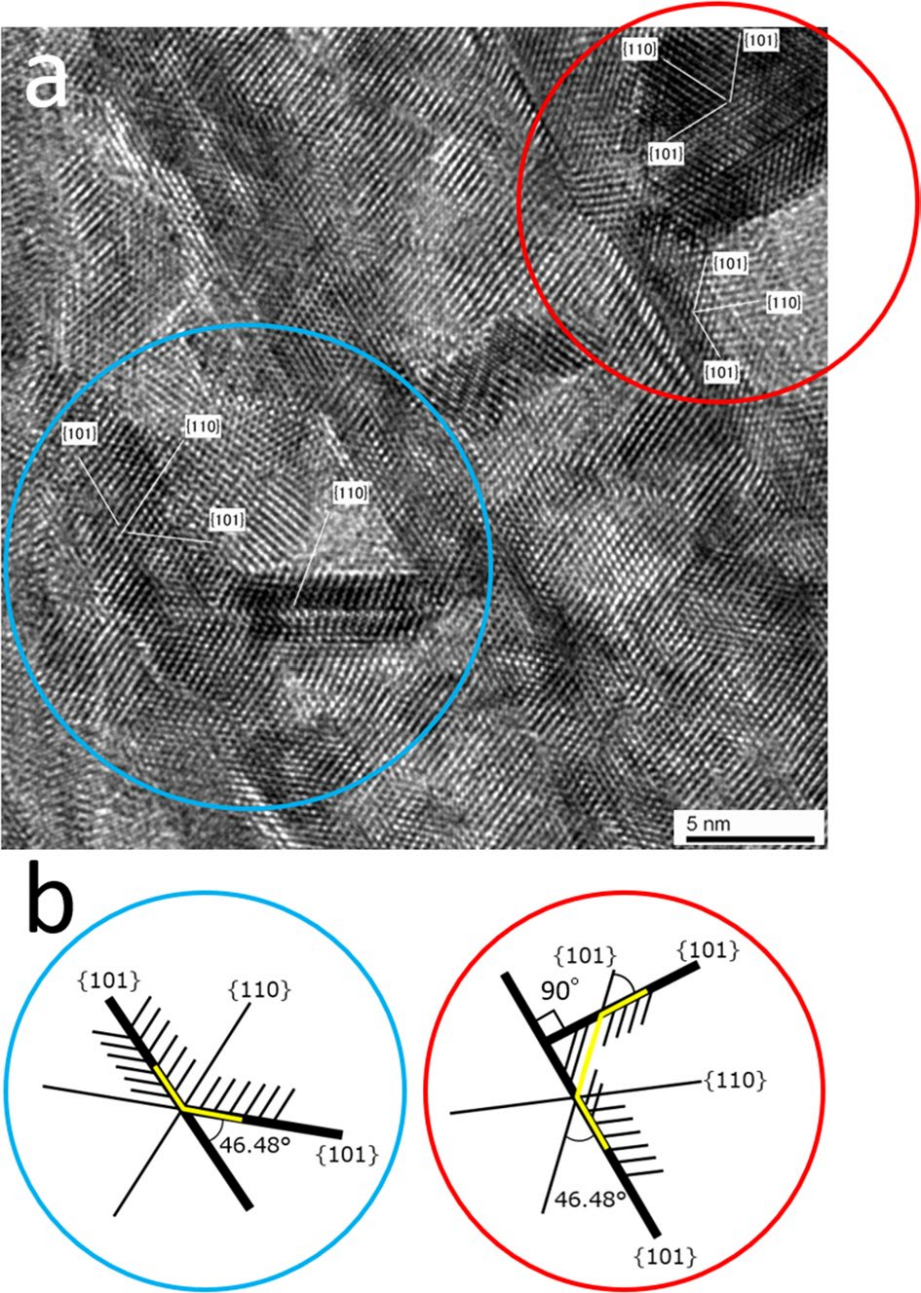
(**a**) High-resolution plane-observed TEM image of the SnO_2_ nanosheets that connected with a branch angle of 90° (square A and B) or 46.48° with notations of the crystal planes. (**b**) Crystal growth models of the SnO_2_ nanosheets that connected with a branch angle of 90° (right, red circle) or 46.48° (left, blue circle). The models were observed from the direction of B =  < 1 − 1 − 1 > of SnO_2_.

The crystal planes of the SnO_2_ nanosheets that connected with a branch angle of 90° or 40°–50° are shown in the red or blue circle, respectively (Fig. 6a). They were analyzed with the FFT images (Fig. 5b) and the calculated SAED pattern (Fig. 5c). The crystal growth models of the SnO_2_ nanosheets that connected with a branch angle of either 90° (red circle) or 46.48° (blue circle) are shown in Fig. 6b. The two bold lines correspond to the {101} crystal planes from the direction of B = <1 − 1 − 1> of SnO_2_ in the red circle (Fig. 6b, right), and their calculated branch angle was 90°. This crystal growth model was based on the branch angle of the type I (90°) connection. Additionally, small nanosheets can grow to connect with the bold lines. A calculated branch angle for that connection was 46.48° (Fig. 6b, right). This crystal growth model was based on the branch angle of the type II (46.48°) connection. The branch angle of 46.48° is clearly shown in the growth model depicted in the blue circle (Fig. 6b, left). The two bold lines correspond to the {101} crystal planes from the direction of B = < 1 − 1 − 1> of SnO_2_, and they connect to form the branch angle of the type II (46.48°) connection.

The branch angle of the type II (46.48°) connection was analyzed with a crystal structure analysis program ReciPro (ver. 4.283). The crystal system, laue group, space group, lattice constant of the *a*-axis, and lattice constant of the *c*-axis were tetragonal, 4/*mmm*, *P*4_2_/*mnm*, 4.737 Å, and 3.185 Å, respectively. The angle between the (101) and (110) crystal planes was 66.76°. The calculated branch angle of the type II connection was 46.48 (= 180 − (66.76 × 2)). The axis normal to both of the (101) and (110) crystal planes was [1 − 1 − 1], which was calculated with the program and consistent with the analysis of the FFT images.

The crystal growth model is summarized as follows. The models that were analyzed with FFT images and the calculated SAED pattern indicted that the crystal growth of the SnO_2_ nanosheet assembled film was based on dendritic growth. The branch angle of the nanosheets was determined by the crystal structure and growth direction. The nanosheets had two types of connections in which the branch angle was 90° (type I) or 46.48° (type II).

### Crystallographic analysis of the SnO_***2***_ (101) nanosheet assembled film with cross-sectional TEM images

**Figure 7:**
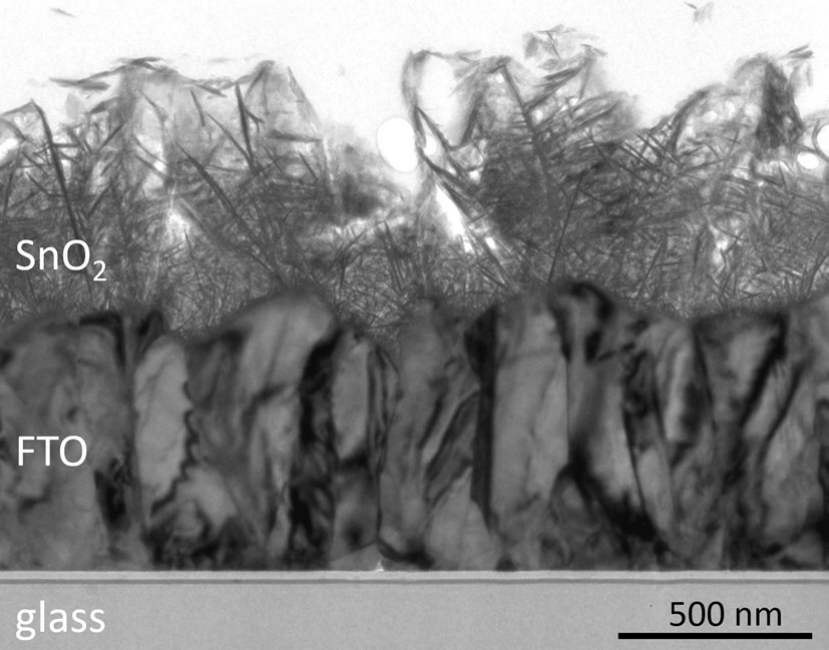
Cross-sectional TEM image of the SnO_2_ (101) nanosheet assembled film.

The SnO_2_ nanosheets were nucleated and grown on the FTO surface to form the SnO_2_ (101) nanosheet assembled film (Fig. 7). The glass substrate, at the bottom of the TEM image, had a flat surface. The glass substrate had a dark gray layer of approximately 10 nm and a light gray layer of approximately 20 nm near the interface with the FTO layer. The brightness of the light gray layer was similar to that of the glass substrate. The FTO layer formed on the glass substrate. The thickness of the FTO layer was 500–800 nm, and the thickness of its rough surface was 100–300 nm. The FTO layer had dark gray and light gray regions and was composed of a polycrystal of SnO_2_. The difference in the crystal axis direction of each crystal caused differences in the brightness. The SnO_2_ (101) nanosheet assembled film formed on the surface of the FTO layer. The film had a porous structure, its density was lower toward the surface, and its thickness was 500–800 nm.

**Figure 8:**
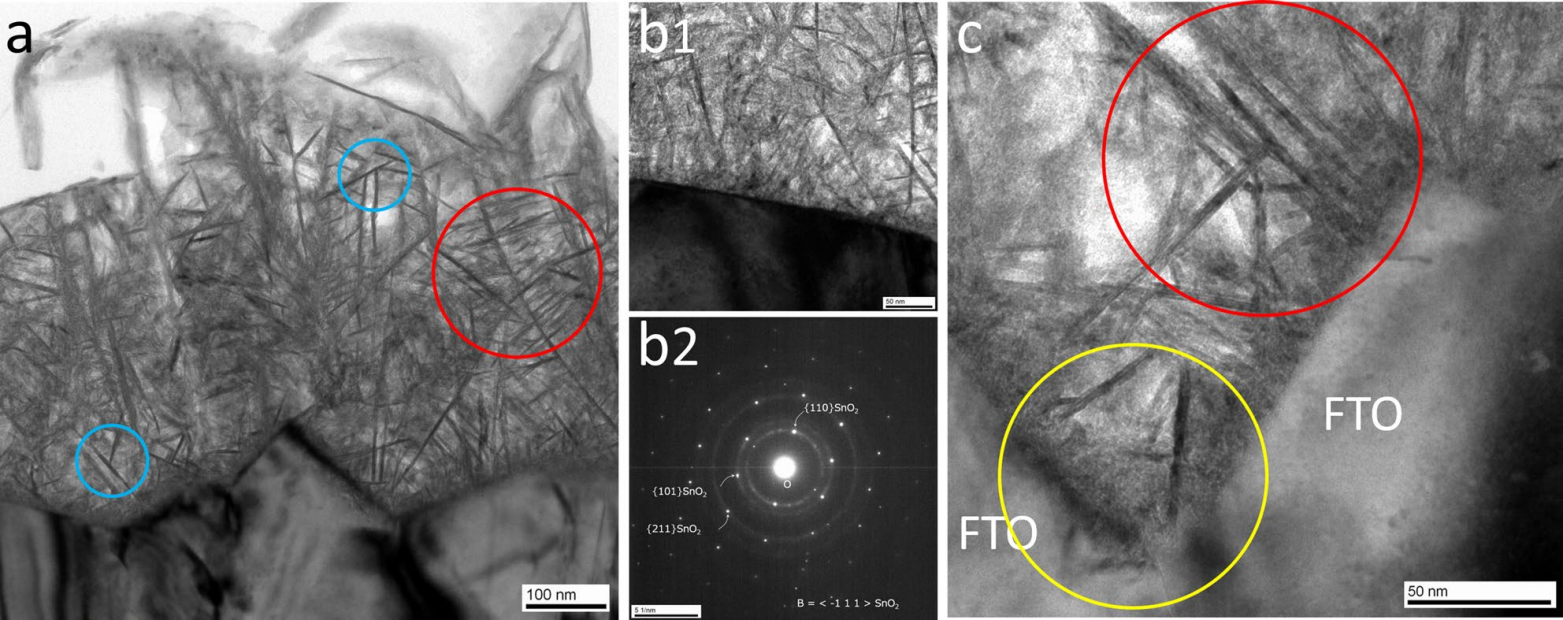
(**a**) Cross-sectional TEM image of the gradient structure of the SnO_2_ (101) nanosheet assembled film on the FTO substrate showing branch angles of 90° (type I) (red circle) and 46.48° (type II) (blue circle) between the SnO_2_ nanosheets. (**b1**) Cross-sectional TEM image of the SnO_2_ (101) nanosheet assembled film on the FTO substrate. (**b2**) SAED pattern from (**b1**) showing the electron diffraction from SnO_2_. (**c**) Cross-sectional TEM image of the SnO_2_ (101) nanosheet assembled film on the FTO surface in the depressed valleys showing type I connections (branch angle of 90°) between the SnO_2_ nanosheets (red circle), and high surface coverage in the concave area (yellow circle).

The nanosheets were small in size and formed a dense structure in the region close to the FTO substrate (Fig. 8a). On the other hand, they were also large in size and formed a porous structure in the region far from the FTO substrate. The film had a gradient structure in the direction perpendicular to the substrate; this gradient structure was one of the characteristics of the film. The nanosheets connected with other nanosheets, i.e., one nanosheet branched and grew from another nanosheet. Many small nanosheets branched and grew from the large nanosheet (red circle, Fig. 8a). Notably, a branch angle of 90° (red circle, Fig. 8a) was observed between the small nanosheet and the large nanosheet. This angle is associated with the type I connection (red circles, Figs. 4a, 6a), and the corresponding model is depicted in the red circle of Fig. 6b. A different branch angle of 46.48° was also observed, as shown in the blue circles of Fig. 8a. This angle is associated with the type II connection (blue circles, Figs. 4b,c, 6a), and the corresponding model is depicted in the blue circle of Fig. 6b.

An SAED pattern was obtained from the interface between the SnO_2_ (101) nanosheet assembled film and the FTO layer (Fig. 8b1). Orderly arrays of strong diffraction spots were observed (Fig. 8b2), and they were assigned to the {110}, {100}, or {200} diffraction planes of the SnO_2_ crystal from the <− 111> direction. A large SnO_2_ crystal of the FTO layer was observed in a TEM image (Fig. 8b1), and it had a flat crystal facet. The large SnO_2_ crystals in the FTO layer caused the clear spots. In addition, Debye rings were observed, and they were assigned to the {110}, {100}, or {200} diffraction planes of the SnO_2_ crystal. The presence of Debye ring diffraction indicated that the rings originated from the random oriented crystals of the SnO_2_ nanosheets.

The branch angle of 90° (type I) was also observed at the interface between the SnO_2_ nanosheet and the FTO layer, shown in the red circle (Fig. 8c). Many SnO_2_ nanosheets were oriented perpendicular to the surface of the FTO layer. The observed angle between any two SnO_2_ nanosheets at the center of the red circle was also 90°. The FTO layer had large surface roughness since the FTO was a SnO_2_ polycrystal. Two crystal surfaces in the FTO layer formed an angle of less than 90° (yellow circle, Fig. 8c). In spite of the surface roughness, the SnO_2_ nanosheets formed on the surface of the depressed valleys to cover the entire surface of the FTO layer. This is one of the advantages of the solution process.

**Figure 9:**
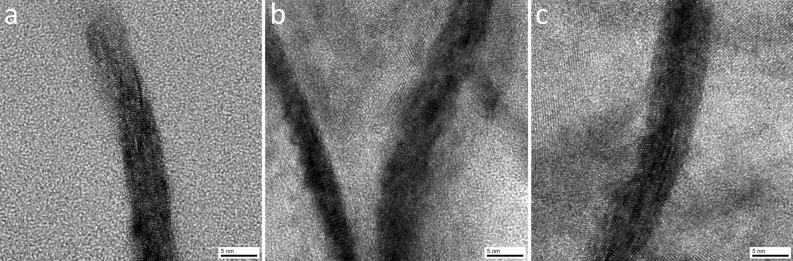
(**a**) Cross-sectional TEM image of the tip of the SnO_2_ nanosheet from a direction parallel to the plane. (**b**,**c**) Cross-sectional TEM image of the middle of the SnO_2_ nanosheets from a direction parallel to the plane.

The SnO_2_ nanosheets were also observed from a direction parallel to the plane (Fig. 9a–c), where the crystal lattice fringes were observed. These surfaces contained no cracks or pores and were not covered with amorphous layers or organic layers. The bare SnO_2_ crystal was exposed even at the tip of the nanosheet (Fig. 9a). The surface of the nanosheet was exposed to the external atmosphere, which led to the characteristic sensor properties. The thickness evaluated at several points was 4–8 nm (Fig. 9a–c); this thinness provided a high specific surface area^20^ and also contributed to the weight reduction of the coating film. In addition, this dimension had the effect of increasing the ratio of the nano space to SnO_2_ crystal. Furthermore, studies showed that the proportion of the electron depletion layer in the SnO_2_ crystal dramatically increased in the semiconductor gas sensor^9–11,19^, which resulted in high sensor sensitivity.

**Figure 10:**
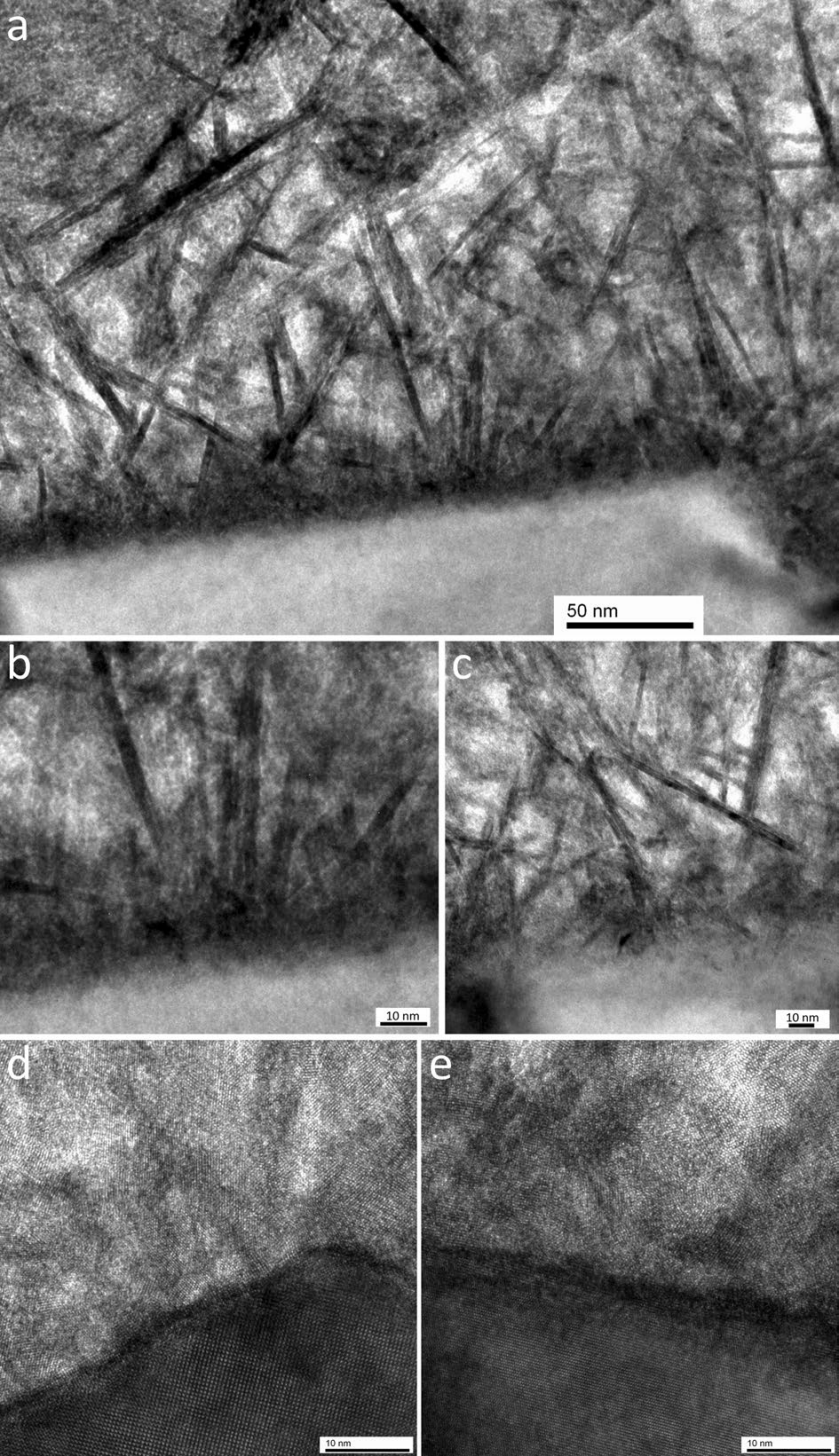
(**a**) Cross-sectional TEM image of the interface between the SnO_2_ nanosheet and the FTO layer. (**b**,**c**) Magnified images showing the crystal growth of the SnO_2_ nanosheets at the initial stage. (**d**,**e**) High-resolution images showing the crystal lattice fringes of the SnO_2_ nanosheets and the FTO layer.

The interface between the SnO_2_ nanosheet and the FTO surface was observed at multiple points. The results indicated that the SnO_2_ nanosheets directly formed on the FTO surface without any clearance space (Fig. 10a), and the FTO surface was uniformly covered with the SnO_2_ nanosheet assembled film even in the convex area of the FTO surface (Fig. 10a). Therefore, direct nucleation and crystal growth of the SnO_2_ nanosheets were realized without a seed layer or a buffer layer, which enables film formation on various substrates^21–23^. The magnified areas show the crystal growth of the SnO_2_ nanosheets at the initial stage (Fig. 10b,c). The nanosheets were 2–5 nm in thickness and 5–20 nm in length in the region of 50 nm from any point at the interface. These small nanosheets formed a dense structure near the interface, indicating that the nucleation density of the SnO_2_ nanosheets was high on the FTO surface. Moreover, the high ion concentration accelerated the nucleation and growth of the SnO_2_ nanosheet at the initial stage of the synthesis, causing a high density of the SnO_2_ nanosheets on the FTO surface. The magnified images show the crystal lattice fringes of the SnO_2_ nanosheets and the FTO layer (Fig. 10d,e). Clearance space, amorphous layers, and cracks were not observed between the SnO_2_ nanosheets and the FTO layer even in the high-resolution images.

Many tilted SnO_2_ nanosheets were observed near the interface (Fig. 10a–e). However, the number of SnO_2_ nanosheets that were tilted to the interface decreased with increasing distance from the interface. The initial nuclei were randomly oriented and thus grew into nanosheets with random orientations. The nanosheets that were tilted to the interface reached another nanosheet. The crystal growth parallel to the interface was suppressed by this attachment to the other nanosheet. On the other hand, the SnO_2_ nanosheets that aligned perpendicular to the interface grew to form large nanosheets since they had enough space to grow in the region far from the interface. This is one of the formation mechanisms for the gradient structure of the SnO_2_ (101) nanosheet assembled film. The other mechanism is the decreasing ion concentration. Sn ions were used for the nucleation and crystal growth of the SnO_2_ nanosheets, and the supersaturation degree gradually decreased as the synthesis progressed. A large number of SnO_2_ nanosheets were formed during the initial stage, but the rate of nanosheet formation decreased as the synthesis progressed. Therefore, the depleted ion concentration and reduced supersaturation degree of the solution prevented the formation of the SnO_2_ nuclei at the late stage. The crystal growth of the SnO_2_ nanosheets progressed to form large nanosheets in a dilute solution, and the color of the solution changed with time in support of this mechanism. Specifically, the solution was opaque as the SnO_2_ nanosheet-assembled particles nucleated at the initial stage of the synthesis and gradually became transparent with time. Variations of the SnO_2_ nanosheet-assembled particles with synthesis time has been reported^24^. In particular, the particles changed their morphology, color, surface area, X-ray diffraction pattern, chemical composition, X-ray photoelectron spectra, etc. with synthesis time, which were caused by the gradual changes in the solution condition.

**Figure 11:**
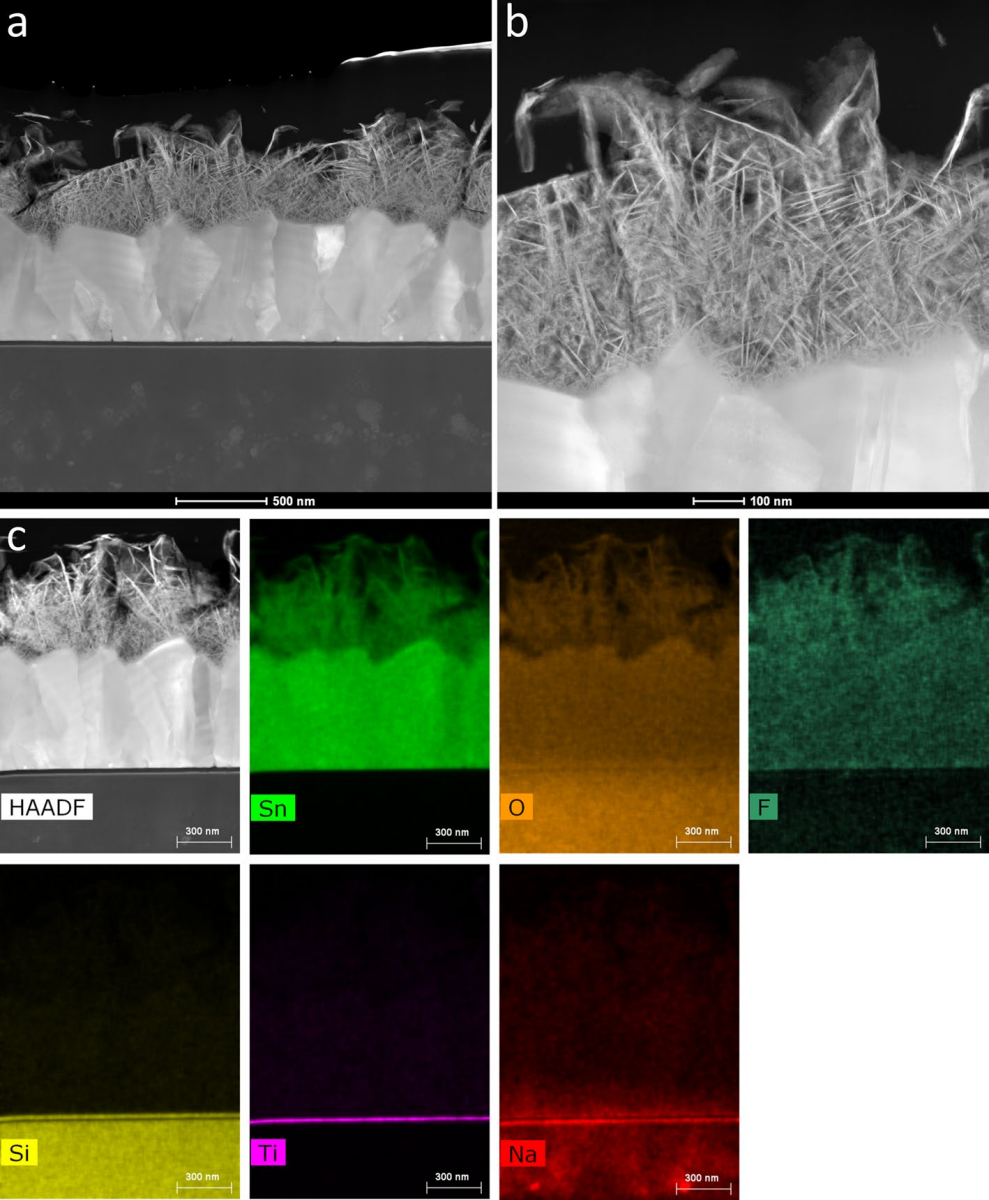
(**a**) Cross-sectional HAADF-STEM image of the SnO_2_ (101) nanosheet assembled film on the FTO substrate. (**b**) Magnified area of (**a**). (**c**) HAADF-STEM image and its EDS mapping images for Sn, O, F, Si, Ti, and Na.

HAADF-STEM images provide contrast information according to factors that affect electron scattering such as sample density, sample thickness, and composition (atomic number) (Fig. 11a). The boundaries of each SnO_2_ crystals in the FTO layer were clearly shown. Cracks and impurities near the interface between the FTO layer and the glass substrate were not observed. A black layer of approximately 23 nm in width and a gray layer of 13 nm in width were observed at the interface between the FTO layer and the glass substrate. The glass substrate included light gray spherical areas of 20–200 nm in size, which had a chemical composition and density that was different from the surroundings. The spherical impurity areas were not observed near the interface of approximately 200 nm in width.

The SnO_2_ (101) nanosheet assembled film in Fig. 8a was observed with HAADF-STEM (Fig. 11b). The shape of the SnO_2_ nanosheets and their connections were clearly observed. The SnO_2_ nanosheets and the FTO layer were similar in opacity (white) compared with the glass substrate since they had similar chemical compositions. The nanosized spaces that were surrounded by the nanosheets were also clearly observed in the SnO_2_ nanosheet assembled film.

Tin was detected in both the SnO_2_ (101) nanosheet assembled film and the FTO layer (Fig. 11c, Sn). Oxygen was observed in the SnO_2_ (101) nanosheet assembled film, the FTO layer, and the glass substrate, while it was not observed in the gray layer of 13 nm (Fig. 11c, O). A similar signal intensity for fluorine was detected in the SnO_2_ (101) nanosheet assembled film and the FTO layer (Fig. 11c, F). The FTO layer was doped with fluorine to obtain high electrical conductivity. Therefore, the SnO_2_ (101) nanosheet assembled film contained a fluorine concentration similar to that in the FTO layer. Silicon was observed in the glass substrate and the black layer of approximately 23 nm in width since the glass contained silicon dioxide (Fig. 11c, Si). The gray layer of 13 nm in width contained titanium (Fig. 11c, Ti). Sodium was detected in the glass (Fig. 11c, Na) and was also detected in the FTO layer near the glass. Therefore, sodium was slightly distributed to the FTO layer from the glass. A high concentration of sodium was observed in the glass near the gray layer of 13 nm in width. Spherical areas with a high concentration of sodium were observed in the glass substrate; one such area overlapped with the scale bar and the other area was below the label of sodium (Fig. 11c, Na). Their positions were the same as the light gray spherical areas in the HAADF-STEM image (Fig. 11c, HAADF), indicating that the light gray spherical impurity areas in the HAADF-STEM images corresponded to the glass with a high concentration of sodium. The titanium layer of 13 nm in width suppressed the diffusion of sodium from the glass into the FTO layer, although sodium was slightly detected in the FTO layer.

### Discussions on the mechanism of anisotropic crystal growth for the nanosheet

#### Surface energy calculations for the most stable (110) facet and metastable (101) facet

SnO_2_ crystal structures with (110) (Fig. S1, left) or (101) (Fig. S1, right) facet were calculated using the VESTA program^25^ with the crystal structure data of SnO_2_ (COD ID:1000062, The Crystallography Open Database)^26^. The oxygen ions were bonded to the tin ions to produce a bridge structure in the outermost surface layer of the (101) facet. They can be easily removed^11^. The surface reactivity is strongly associated with surface energy and surface stability, and the metastable surface is unstable and highly reactive.

The morphology and facets of the SnO_2_ crystals have been discussed with surface energy calculations. Batzill et al*.*^27^ reported a photograph of a SnO_2_ single crystal of approximately 5 mm in size, which was grown by a vapor phase transport reaction^28^. The natural growth faces were the {110}, {100}(equivalent to {010} in rutile), and {101}(equivalent to {011} in rutile) surfaces. Beltran et al*.* calculated the surface energy of stoichiometric SnO_2_ facets with density functional theory using Becke’s three-parameter exchange functional combined with the Lee–Yang–Parr correlation functional (B3LYP) and periodic slab models^29^. They concluded that the (110) facet was the most stable. The surface energies for the (110), (100), (101), (201), and (001) facets were 1.20, 1.27, 1.43, 1.63, and 1.84 J m^−2^, respectively^29^. The surface energy per crystal facet increased in the order (110) < (100) < (101) < (201) < (001). Oviedo et al*.* studied the energetics and the relaxed ionic positions of several low-index stoichiometric SnO_2_ surfaces by first-principles calculations based on the density functional theory, ultrasoft pseudopotentials, and plane-wave basis sets^30^. The generalised gradient approximation (GGA) was used for the calculations. The surface energies for the (110), (100), (101), and (001) facets were 1.04, 1.14, 1.33, and 1.72 J m^−2^, respectively^30^. The surface energy per crystal face increased in the order (110) < (100) < (101) < (001). They concluded that the (110) surface was the most stable from an electrostatic perspective because it had the lowest density of dangling bonds. Mulheran et al*.* calculated the surface energy of SnO_2_ using local-density approximation (LDA)^31^. The surface energies for the (110), (210), (101), (100), (310), (321), (211), (301), (111), and (001) facets were 1.380, 1.487, 1.554, 1.664, 1.679, 1.758, 1.821, 1.860, 2.217, and 2.366 J m^−2^, respectively^31^. The surface energy per crystal face increased in the order (110) < (210) < (101) < (100) < (310) < (321) < (211) < (301) < (111) < (001). They also reported that the calculated excess surface energies per unit cell were dominated by the electrostatic contribution and were ordered in the same sequence as the areas of the surface unit cells. In contrast, the thermodynamic quantity of excess energy per unit area was dominated by the relaxation energy of the surfaces and did not follow any simple ordering. The authors discussed the implications of these findings for the oxide applications in sensors. Slater et al*.* reported the results of surface energy calculations using LDA^32^. The surface energies for the (110), (210), (101), (100), (310), (321), (211), (301), (311), (111), (001), (212), (221), and (112) facets were 1.401, 1.480, 1.554, 1.648, 1.973, 1.731, 2.135, 1.824, 2.051, 2.209, 2.363, 2.351, 2.280, and 3.677, respectively. The surface energy per crystal face increased in the order (110) < (210) < (101) < (100) < (310) < (321) < (211) < (301) < (311) < (111) < (001) < (212) < (221) < (112).

These calculations indicated that the (110) surface was the most thermodynamically stable and expected to feature predominantly in the morphology. However, the (101) surface was metastable with a higher surface energy, consistent with the observation of stable surfaces of SnO_2_ nanoribbons^29^. The nanoribbons were grown by a controlled carbothermal reduction process, where they grew along the [101] crystal direction and had a rectangular cross section. The (110) facet was identified as the predominant surface in the polycrystalline SnO_2_ nanoribbons. The theoretical calculations were also consistent with the stable surfaces of SnO_2_ nanorods. Vayssieres et al*.* reported on highly ordered SnO_2_ nanorod arrays^33^ in which the nanorods grew along the *c*-axis to form stable prismatic (110) faces. The nanorods had a square cross section and were polycrystalline. They consisted of bundles of finer nanorods of 2–4 nm in width (aspect ratio of about 1:100). The lattice spacings of 0.33, 0.235, and 0.16 nm were indexed to the (110), (111), and (002) facets of rutile SnO_2_, respectively. The nanorods were covered with side (110) faces and top (001) faces.

#### Mechanism of anisotropic growth of the SnO_2_ (101) nanosheet with facet controlled growth method

The mechanism of the anisotropic growth of the SnO_2_ nanosheet is proposed as follows. The (110) crystal facets are the most stable facet, as discussed above. They are also highly stable to etching by fluorine ions in SnF_2_ solutions. Therefore, SnO_2_ grows along the direction perpendicular to the (110) crystal plane into stacked (110) crystal planes. On the other hand, the (101) crystal facets are metastable. A surface zig-zag structure of Sn–O–Sn in which the oxygen ions are at the outermost surface decreases the chemical stability of SnO_2_. The bridging oxygen ions are unstable and easy to remove. The (101) crystal facets are etched by the fluorine ions in the SnF_2_ solution. Both etching and growth occur on the (101) crystal facet to achieve slow crystal growth of SnO_2_ along the direction perpendicular to the (101) crystal facet to produce stacked (101) crystal planes. The difference in the crystal growth speed along the direction perpendicular to the (110) crystal facet and the (101) crystal facet causes anisotropic growth to form the nanosheet with large (101) crystal facets.

Precise control of the balance between crystal growth and dissolution can be realized with the proposed facet controlled growth method. Therefore, crystals with metastable crystal facets can be obtained. The anisotropic crystal growth by this method can be applied to various crystal morphology control. An etching agent other than fluorine ions can be also used for etching. In addition, etching can be performed by a change in pH, temperature, raw material, concentration ratio, additives, etc. It can be utilized for anisotropic growth control and morphology control of various crystals including inorganic crystals, organic crystals, biocrystals, metal organic frameworks, etc. to facilitate the development of devices using novel crystal planes and morphologies.

Experimental studies are available to support the mechanism of anisotropic crystal growth for the nanosheet mentioned above. Choi et al*.* revealed that there were many oxygen vacancies (V_o_^··^) and non-stoichiometric Sn^2+^ on the (101) facet of the SnO_2_ nanosheets compared with the (110) facet of the SnO_2_ nanoparticles using X-ray photoelectron spectroscopy (XPS)^11^. The oxygen vacancies indicated that the bond strength between oxygen and tin on the (101) facet was low. In addition, it was suggested that oxygen ions on the (101) facet were easily released and etched. The ratio of the O component bound to Sn^2+^ to the O component bound to Sn^4+^ was 0.1% for the nanoparticles and 9.7% for the nanosheet. The unstable Sn^2+^ site on the (101) facet was easily etched and was one of the sites of etching. In addition, the presence of Sn^2+^ indicated the presence of oxygen vacancies. They also reported that the oxygen component ratio of lattice-Sn^2+^:lattice-Sn^4+^:deficiency:chemisorption was 4.3:43.8:30.0:21.9 for the nanosheets and 0.0:7.4:1.4:91.2 for the nanoparticles. The ratio recalculated with the total of lattice-Sn^2+^, lattice-Sn^4+^, and deficiency normalized to 100 was 5.5:56.1:38.4 for the nanosheets and 0.0:84.1:15.9 for the nanoparticles. Therefore, the (101) facet of the nanosheet contained 2.4 times (= 38.4/15.9) more of the stronger oxygen peak component due to oxygen deficiency than the (110) facet of the nanoparticles. The oxygen vacancies caused crystal imperfections and weakened the Sn–O bonds on the (101) facet of the nanosheet, thereby accelerating the etching of the (101) facet. The (110) facet of the nanoparticles contained 4.1 times more of the stronger oxygen peak component due to chemisorption than the (101) facet of the nanosheet. Therefore, the nanoparticles were covered with an adsorbed oxygen layer, which acted as a barrier to protect the oxygen and tin ions of the (110) facet. This protective effect that suppresses the release of oxygen from the (110) facet contributes to stability.

High etching speed of the unstable surface was also observed on TiO_2_, which has the same rutile crystal structure as SnO_2_^34^. The converged surface energies were calculated from model systems using GGA and three pseudopotentials^35^. The surface energies for the (110), (100), (101), and (001) facets calculated with US10 were 0.54, 0.76, 1.08, and 1.32 J m^−2^, respectively. The surface energies for the (110), (100), (101) or (001) facets calculated with PAW4 were 0.50, 0.69, 1.03, and 1.25 J m^−2^, respectively. The surface energies for the (110), (100), (101), and (001) facets calculated with PAW10 were 0.48, 0.67, 1.01, and 1.21 J m^−2^, respectively. Oviedo et al*.* also reported the results of surface energy calculations^30^. The surface energies for the (110), (100), (101), and (001) facets calculated with LDA were 0.89, 1.12, 1.39, and 1.65 J m^−2^, respectively. They indicated that the surface energy the different facets of rutile TiO_2_ increased in the order (110) < (100) < (101) < (001). This trend was similar to that of SnO_2_ due to the similarity in their crystal structure. Rutile TiO_2_ nanorods were reported to possess a (001) core and (110) sidewall facet^34^. The TiO_2_ nanorod was etched in a hydrothermal solution containing 9 mL of HCl (36.5–38 wt%) and 7 mL of deionized water at 150 °C for 4–6 h, and the metastable (001) facet of the core was observed to etch faster than the most-stable (110) facet of the sidewall.

#### Calculated model of the SnO_***2***_ nanosheet with large (101) and (− 10− 1) facets

**Figure 12:**
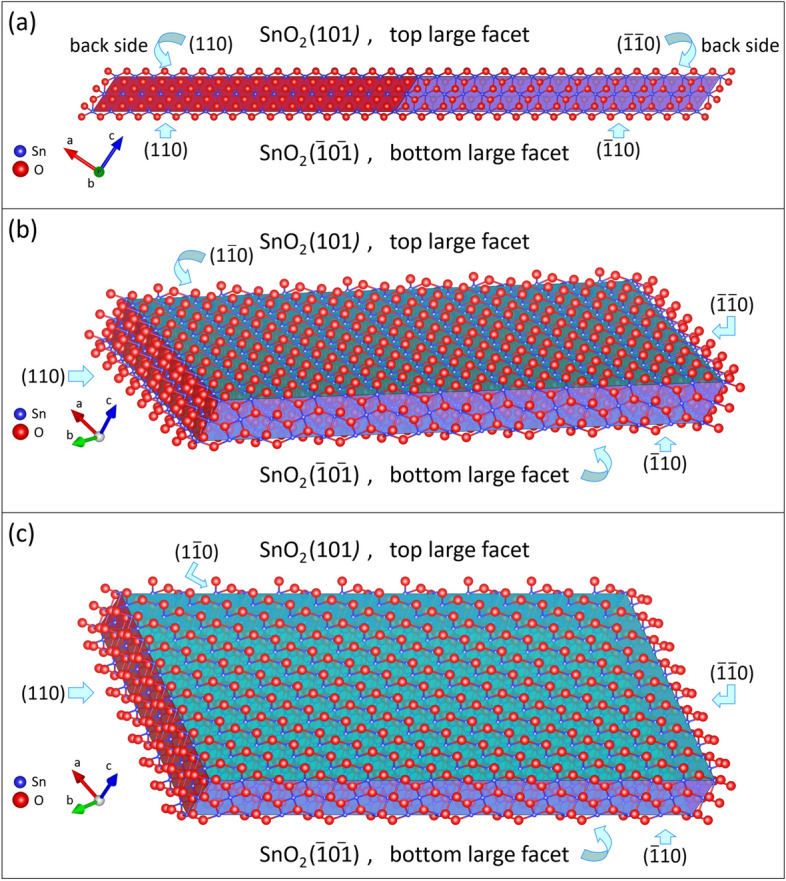
(**a**) Model of the SnO_2_ nanosheet with large (101) and (− 10− 1) facets. (**b**,**c**) Tilted images of the SnO_2_ nanosheet. Calculations were performed using the VESTA program^25^ and crystal structure data of SnO_2_ (COD ID:1000062)^26^.

The nanosheet with large (101) and (− 10− 1) facets was modeled with VESTA (Fig. 12a). These two facets were parallel and equivalent and contained surface bridging oxygen ions. The tilted image showed four facets on the sides of the nanosheet (Fig. 12b), which were the equivalent most-stable facets of (110), (− 110), (− 1− 10), and (1− 10). A further tilted image showed the appearance of the nanosheet with two large metastable (101) facets and four narrow and most-stable (110) facets (Fig. 12c).

The fluorine ions in hydrofluoric acid are known to dissolve metal oxides such as SiO_2_ or SnO_2_ by cleaving the chemical bonds between a metal ion and an oxygen ion. The unstable (101) facets are easily etched with fluorine ions compared with the most-stable (110) facets, and the bridging oxygens are also easily attacked by fluorine ions. Thus, fluorine ions continuously dissolve the (101) facet while the tin ions in the solution stack the (101) atomic layers to promote crystal growth perpendicular to the (101) facet. The crystal growth rate in the direction perpendicular to the (101) facet is determined by the competing dissolution rate determined by the fluorine ions and growth rate determined by the tin ions. The controlled crystal growth rate in the direction perpendicular to the (101) facet was very slow under the conditions in this study. On the other hand, the dissolution rate of the most-stable (110) facet caused by the fluorine ions was low. Therefore, the crystal growth rate in the direction perpendicular to the (110) facet was maintained at a high rate to balance the dissolution rate determined by the fluorine ions and the growth rate determined by the tin ions. The rate of crystal growth perpendicular to the (101) facet was much slower than that perpendicular to the (110) facet due to the difference in stability between the two facets. This difference in growth rate between the (101) facets and (110) facets resulted in the formation of nanosheets with large (101) facets.

## Conclusion

The SnO_2_ (101) nanosheet assembled film was developed in an aqueous solution. The nanosheets grew directly on the FTO substrate. The thickness of the SnO_2_ (101) nanosheet assembled film was approximately 800 nm. The nanosheets had a thickness of 5–10 nm, an in-plane size of 100–1600 nm, and a metastable (101) crystal facet with a large flat surface.

The crystal growth model was discussed using TEM images, FFT images, and calculated SAED patterns. Dendritic growth governed the crystal growth of the SnO_2_ (101) nanosheet assembled film in which the branch angle of the connected nanosheets was determined by the crystal structure and growth direction, resulting in two types of connections, which were defined by the branch angle of either 90° (type I) or 46.48° (type II). Dendritic growth of the branch structures enabled the formation of the SnO_2_ (101) nanosheet assembled film, which possessed a characteristic network of the nanosheets with a high surface area and internal nano spaces.

Furthermore, the mechanism of anisotropic crystal growth of the nanosheet was discussed. The (110) crystal facets were the most stable and possessed high stability toward etching by the fluorine ions in the SnF_2_ solution. Therefore, SnO_2_ grew along the direction perpendicular to the (110) crystal plane to form stacked (110) crystal planes. On the other hand, (101) crystal facet was metastable, and thus both etching and growth occurred on the (101) crystal facet in the SnF_2_ solution, thereby realizing slow crystal growth along the direction perpendicular to the (101) crystal facet. The facet controlled growth method achieved anisotropic crystal growth to produce the nanosheet with the large (101) facet. This method can be applied to control the morphology of crystals with metastable facets. In conclusion, the characteristic activity of metastable facets is expected to contribute to the development of various devices.

## Supplementary Information

Supplementary Information 1.

## References

[1] Shaikh SF (2018). Low-temperature ionic layer adsorption and reaction grown anatase TiO_2_ nanocrystalline films for efficient perovskite solar cell and gas sensor applications. Sci. Rep..

[2] Zhou C (2014). Ultrasensitive non-enzymatic glucose sensor based on three-dimensional network of ZnO–CuO hierarchical nanocomposites by electrospinning. Sci. Rep..

[3] Filippin AN (2016). Vacuum template synthesis of multifunctional nanotubes with tailored nanostructured walls. Sci. Rep..

[4] Masuda Y, Bekki M, Sonezaki S, Ohji T, Kato K (2009). Dye adsorption characteristics of anatase TiO_2_ film prepared in an aqueous solution. Thin Solid Films.

[5] Masuda Y (2011). High protein-adsorption characteristics of acicular crystal assembled TiO(2) films and their photoelectric effect. Thin Solid Films.

[6] Masuda Y, Ohji T, Kato K (2013). Water bath synthesis of Tin oxide nanostructure coating for a molecular sensor. J. Nanosci. Nanotechnol..

[7] Masuda Y, Ohji T, Kato K (2012). Tin oxide nanosheet assembly for hydrophobic/hydrophilic coating and cancer sensing. ACS Appl. Mater. Interfaces.

[8] Masuda Y, Itoh T, Shin W, Kato K (2015). SnO_2_ nanosheet/nanoparticle detector for the sensing of 1-nonanal gas produced by lung cancer. Sci. Rep..

[9] Choi PG, Izu N, Shirahata N, Masuda Y (2018). Fabrication and H2-sensing properties of SnO_2_ nanosheet gas sensors. ACS Omega.

[10] Choi PG, Izu N, Shirahata N, Masuda Y (2019). SnO_2_ nanosheets for selective alkene gas sensing. ACS Appl. Nano Mater..

[11] Choi PG, Izu N, Shirahata N, Masuda Y (2019). Improvement of sensing properties for SnO_2_ gas sensor by tuning of exposed crystal face. Sens. Actuators B Chem..

[12] Zakaryan H, Aroutiounian V (2017). CO gas adsorption on SnO_2_ surfaces: Density functional theory study. Sens. Transducers.

[13] Jiang C, Zhang G, Wu Y, Li L, Shi K (2012). Facile synthesis of SnO_2_ nanocrystalline tubes by electrospinning and their fast response and high sensitivity to NOx at room temperature. CrystEngComm.

[14] Abokifa AA, Haddad K, Fortner J, Lo CS, Biswas P (2018). Sensing mechanism of ethanol and acetone at room temperature by SnO_2_ nano-columns synthesized by aerosol routes: Theoretical calculations compared to experimental results. J. Mater. Chem. A.

[15] Feng H (2017). Three-dimensional hierarchical SnO_2_ dodecahedral nanocrystals with enhanced humidity sensing properties. Sens. Actuators B Chem..

[16] Dressick WJ, Calvert JM (1993). Patterning of self-assembled films using lithographic exposure tools. Jpn. J. Appl. Phys..

[17] Dressick WJ, Dulcey CS, Georger JH, Calvert JM (1993). Photopatterning and selective electroless metallization of surface-attached ligands. Chem. Mater..

[18] Masuda Y (2020). Bio-inspired mineralization of nanostructured TiO_2_ on PET and FTO films with high surface area and high photocatalytic activity. Sci. Rep..

[19] Choi PG, Shirahata N, Masuda Y (2020). Tin oxide nanosheet thin film with bridge type structure for gas sensing. Thin Solid Films.

[20] Masuda Y, Kato K (2009). Aqueous synthesis of nano-sheet assembled tin oxide particles and their N_2_ adsorption characteristics. J. Cryst. Growth.

[21] Masuda Y, Kato K (2010). Tin oxide coating on polytetrafluoroethylene films in aqueous solutions. Polym. Adv. Technol..

[22] Masuda Y, Ohji T, Kato K (2011). Site-selective chemical reaction on flexible polymer films for tin oxide nanosheet patterning. Eur. J. Inorg. Chem..

[23] Masuda Y, Kato K (2012). SnO_2_ nanosheet assembly coating on teflon rods. J. Aust. Ceram. Soc..

[24] Masuda Y (2012). Crystal growth of tin oxide nano-sheets in aqueous solutions and time variation of N2 adsorption characteristics. Prog. Cryst. Growth Charact. Mater..

[25] Momma K, Izumi F (2011). VESTA 3 for three-dimensional visualization of crystal, volumetric and morphology data. J. Appl. Crystallogr..

[26] Baur WH, Khan AA (1971). Rutile-type compounds. IV. SiO_2_, GeO_2_ and a comparison with other rutile-type structures. Acta Crystallogr. Sect. B.

[27] Batzill M (2005). Gas-phase-dependent properties of SnO_2_ (110), (100), and (101) single-crystal surfaces: Structure, composition, and electronic properties. Phys. Rev. B.

[28] Park PW, Kung HH, Kim DW, Kung MC (1999). Characterization of SnO_2_/Al_2_O_3_ lean NOx catalysts. J. Catal..

[29] Beltrán A, Andrés J, Longo E, Leite ER (2003). Thermodynamic argument about SnO_2_ nanoribbon growth. Appl. Phys. Lett..

[30] Oviedo J, Gillan MJ (2000). Energetics and structure of stoichiometric SnO_2_ surfaces studied by first-principles calculations. Surf. Sci..

[31] Mulheran PA, Harding JH (1992). The stability of SnO_2_ surfaces. Modell. Simul. Mater. Sci. Eng..

[32] Slater B, Catlow CRA, Gay DH, Williams DE, Dusastre V (1999). Study of surface segregation of antimony on SnO_2_ surfaces by computer simulation techniques. J. Phys. Chem. B.

[33] Vayssieres L, Graetzel M (2004). Highly ordered SnO_2_ nanorod arrays from controlled aqueous growth. Angew. Chem. Int. Ed..

[34] Wan J (2016). Hydrothermal etching treatment to rutile TiO_2_ nanorod arrays for improving the efficiency of CdS-sensitized TiO_2_ solar cells. Nanosc. Res. Lett..

[35] Perron H (2007). Optimisation of accurate rutile TiO_2_ (110), (100), (101) and (001) surface models from periodic DFT calculations. Theoret. Chem. Acc..

