# Supplementary material for: Facet controlled growth mechanism of SnO2 (101) nanosheet assembled film via cold crystallization
Source: Sci Rep. 2021 May 28;11:11304. doi: 10.1038/s41598-021-90939-4 (PMC8163760; doi:10.1038/s41598-021-90939-4)
Supplement: Supplementary file 1 — Supplementary Information 1. [file 41598_2021_90939_MOESM1_ESM.docx]

**Supplementary Information**

**Facet controlled growth mechanism of SnO_2_ (101) nanosheet assembled film via cold crystallization**

Yoshitake Masuda*

National Institute of Advanced Industrial Science and Technology (AIST), 2266-98 Anagahora, Shimoshidami, Moriyama-ku, Nagoya 463-8560, Japan

***Corresponding author.** E-mail: masuda-y@aist.go.jp

***Calculation of the SnO_2_ crystal structures with (110) or (101) facet***

SnO_2_ crystal structures with (110) or (101) facet were calculated using the VESTA program^1^ (Fig. S1). The crystal structure data of SnO_2_ (COD ID:1000062)^2^ was introduced from the Crystallography Open Database. The system, Hermann-Mauguin symmetry space group, number of space group, *a*-axis lattice constant, *c*-axis lattice constant, alpha angle, beta angle, gamma angle, unit cell volume, and Sn ion radius and O ion radius of SnO_2_ were tetragonal, *P*4_2_/*mnm*, No. 136, 4.738 Å, 3.1865 Å, 90°, 90°, 90°, 71.5 Å^3^, 0.69 Å or 1.40 Å, respectively. Both the SnO_2_ crystal structures with either the (110) facet (Fig. S1, left) or (101) facet (Fig. S1, right) were in the stoichiometric state. The tin ion was sixfold-coordinated and the oxygen ion was threefold-coordinated in the crystal. The (110) facet had two types of surface oxygen. Oxygen in the atmosphere can be adsorbed at the tin position on the surface. The outermost surface layer of the (110) facet had sixfold-coordinated tin ions, fivefold-coordinated tin ions, in-plane oxygen ions, and bridging oxygen ions. On the other hand, the oxygen ions were bonded to the tin ions to produce a bridge structure in the outermost surface layer of the (101) facet. The second layer contained fivefold-coordinated tin ions. The third and fourth layer contained stable threefold-coordinated oxygen ions and sixfold-coordinated tin ions, respectively. The outermost surface oxygen ions had an unstable bridge structure. The outermost surface tin ions had unstable coordination numbers that were different from those in the crystal. The oxygen ions with a bridge structure can be easily removed^3^. The surface reactivity is strongly associated with surface energy and surface stability, and the metastable surface is unstable and highly reactive.


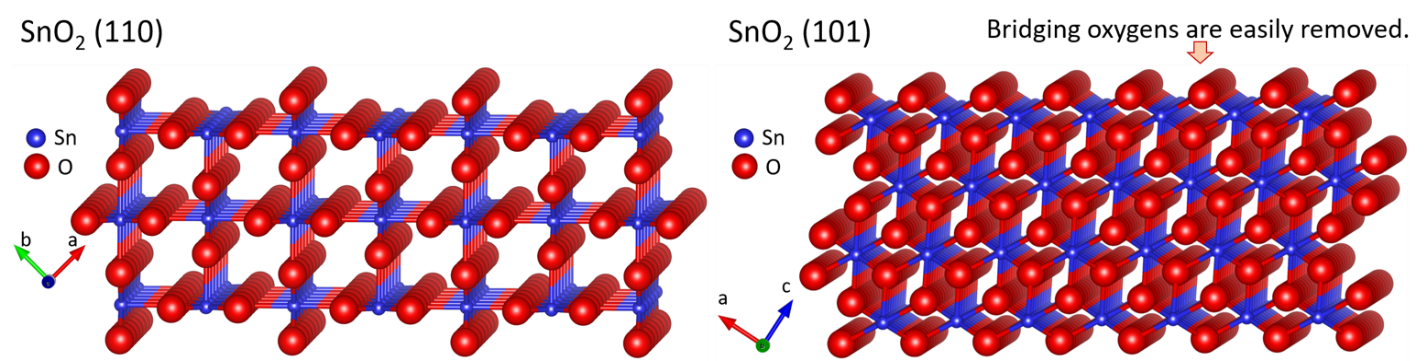


Figure S1. (left) Model of the SnO_2_ crystal structure with the (110) facet. (right) Model of the SnO_2_ crystal structure with the (110) facet. Calculations were performed using the VESTA program^1^ and crystal structure data of SnO_2_ (COD ID:1000062)^2^.

References

1 Momma, K. & Izumi, F. VESTA 3 for three-dimensional visualization of crystal, volumetric and morphology data. *Journal of Applied Crystallography* **44**, 1272-1276, doi:doi:10.1107/S0021889811038970 (2011).

2 Baur, W. H. & Khan, A. A. Rutile-type compounds. IV. SiO_2_, GeO_2_ and a comparison with other rutile-type structures. *Acta Crystallographica Section B* **27**, 2133-2139, doi:10.1107/s0567740871005466 (1971).

3 Choi, P. G., Izu, N., Shirahata, N. & Masuda, Y. Improvement of sensing properties for SnO_2_ gas sensor by tuning of exposed crystal face. *Sensors and Actuators B: Chemical* **296**, 126655, doi:<https://doi.org/10.1016/j.snb.2019.126655> (2019).
